# Microdroplet Mass Spectrometry Enables Extremely Accelerated Pepsin Digestion of Proteins

**DOI:** 10.1021/jasms.1c00126

**Published:** 2021-06-08

**Authors:** Tobias Rainer, Reiner Eidelpes, Martin Tollinger, Thomas Müller

**Affiliations:** Institute of Organic Chemistry and Center for Molecular Biosciences (CMBI), Leopold-Franzens University Innsbruck, 6020 Innsbruck, Austria

**Keywords:** mass spectrometry, peptides and proteins, pepsin digestion, microdroplet reaction acceleration

## Abstract

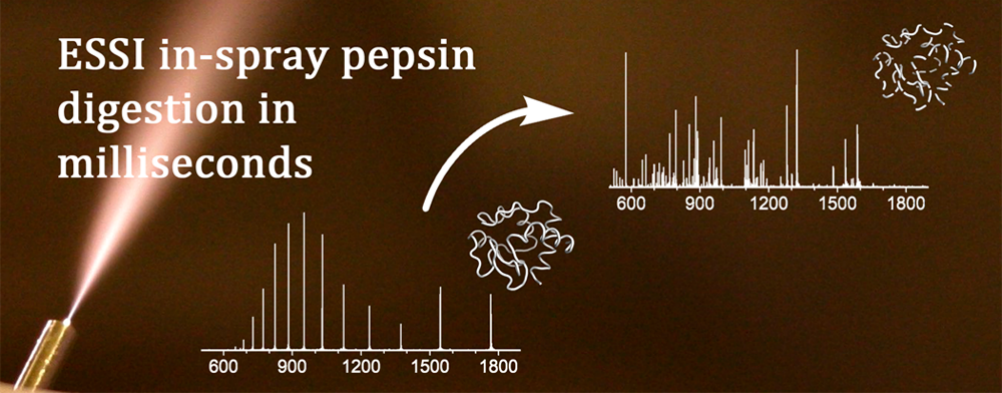

In microdroplets, rates of chemical or biomolecular reactions can exceed those in the bulk phase by more than a million times. As electrospray ionization-based mass spectrometry (MS) involves the formation of charged microdroplets, reaction acceleration and online MS monitoring of reaction products can readily be performed at the same time. We investigated accelerated enzymatic reactions in microdroplets and focused on the proteolytic enzyme pepsin. Electrosonic spray ionization (ESSI) was utilized for developing the ultrarapid pepsin in-spray digestion of two different proteins, cytochrome *c* and RocC, at low pH values. The optimization of the protein digestion aimed at achieving maximum sequence coverage for the analyzed proteins. Furthermore, carefully designed control experiments allowed us to unambiguously prove that enzymatic protein cleavage almost exclusively occurs within the spray at a millisecond time scale and not prior to microdroplet generation.

## Introduction

Rapid developments in the field of electrospray-based ambient MS^1^ have paved the way to a variety of novel applications of mass spectrometry.^2^ In particular, the phenomenon of reaction acceleration in airborne charged microdroplets attracts researchers from various backgrounds.^3−26^ Compared to chemical reactions in the bulk phase, rates of reactions in microdroplets were found to be increased by several orders of magnitude, often showing exceptionally high yields. Applications are the accelerated organic synthesis in preparative electrospray,^3−5^ the determination of protein unfolding kinetics, H/D exchange for metabolomic labeling,^6^ and ultrafast biomolecular reactions in microdroplets.^7^ Some reactions in microdroplets even yield reaction products that are not observed under liquid bulk conditions,^8^ or reactions occur without the presence of catalysts, which are required under bulk conditions.^5,9,10^ Many means of reaction acceleration have been studied. However, the extreme increase in reaction rates in microdroplets—in some cases, up to more than a million times compared to bulk reaction rates—is unparalleled.^11^

Reaction acceleration in microdroplets has mainly been attributed to the special chemical and physical conditions inside the confined microscopic reaction vessels,^12,13^ for example, surface excess charge accumulation,^14^ extreme pH,^15^ and/or surface concentration values^16^ and rapid continuous desolvation effects.^17^ While increasing efforts have been made to elucidate the underlying principles of reaction acceleration in microdroplets, such as quantum mechanical calculations,^18^ they are not fully understood yet.^14,19^

Charged microdroplets are easily produced utilizing ambient MS-related techniques such as desorption electrospray ionization (DESI),^7^ electrospray,^3,5^ microfluidic systems,^20^ droplet casting,^21^ or levitated Leidenfrost droplets.^22^ Electrosonic spray ionization (ESSI) appeared to be particularly useful^3,5,12,23−25^ and was recently utilized for dramatically accelerated biomolecular reactions. Using an ultrafast trypsin digestion,^24^ a series of proteins from oligopeptides to therapeutic monoclonal antibodies with different proteolytic susceptibilities were studied, and even protease-resistant peptides could be cleaved under certain conditions. More recently, the same group applied this technique for antibody characterization by microdroplet digestion, reduction, and deglycosylation.^26^ However, it remains unclear whether a broader variety of enzymatic reactions can be accelerated utilizing microdroplet chemistry.

Although the site-specific protease activity of trypsin is advantageous, tryptic digests have to be performed within a narrow pH range from 7 to 9 for optimal enzymatic activity.^27^ Since our future vision is to utilize enzymatic microdroplet digestion in the context of protein hydrogen–deuterium exchange experiments (HDX), we developed a method for ultrarapid pepsin digestion of proteins in charged microdroplets at low pH levels. In addition, our experimental setup allows to distinguish the negligibly slow bulk reaction in the capillary from the exceptionally accelerated digestion in the charged microdroplets.

## Experimental Section

### Reagents and Chemicals

Pepsin from porcine gastric mucosa (P887), lyophilized powder ≥3200 units·mg^–1^ protein and cytochrome *c* from equine heart (C7752), ≥95%, ammonium acetate for LC-MS LiChropur, and pH test strips were purchased from Sigma-Aldrich (St. Louis, USA). LC-MS grade water, ammonium hydrogen carbonate for LC-MS, acetic acid 100% for LC-MS LiChropur, and formic acid puriss. p.a., ACS reagent, reag. Ph. Eur. ≥98% were obtained from Merck (Darmstadt, Germany). Ammonia aqueous 26% Ph. Eur. Riedel-de Haën was obtained from Sigma-Aldrich (Seelze, Germany). PD-10 prepacked desalting columns were purchased from GE Healthcare (Chicago, USA).

### Preparation of Protein and Protease Solutions

The following aqueous solutions were prepared for the DoE experiments: 20 mM cytochrome *c* in 10 mM NH_4_HCO_3_ (*Cyt1*); 20 mM cytochrome *c* in 5 mM NH_4_HCO_3_ (*Cyt2*); 10 mM cytochrome *c* in 5 mM NH_4_OAc (*Cyt3*); 15 μg·mL^–1^ pepsin in 20% acetic acid (*Pep1*); 30 μg·mL^–1^ pepsin in 20% acetic acid (*Pep2*); 30 μg·mL^–1^ pepsin in 4% formic acid (*Pep3*). All samples were stored in aliquots of 100 μL at −20 °C, and pepsin solutions were prepared the day before use. In-house produced and purified recombinant RocC_24–126_ protein samples were prepared as described by Eidelpes et al.^28^ and desalted using PD-10 prepacked desalting columns (GE Healthcare, Chicago, USA) according to the manufacturer’s protocol. MS grade 5 mM NH_4_HCO_3_ solution was used as the elution buffer, resulting in a 0.26 mg·mL^–1^ protein solution (*RocC1*).

### In-Spray Digestion and Parameter Optimization Using a Design of Experiment (DoE) Approach

A dual syringe pump separately dispensed both protein and protease solutions at equal flow rates. After thorough online mixing using a T-piece and a downstream sintered silica frit, an in-lab built ESSI sprayer was used for directly spraying the reaction mixture at the inlet of the mass spectrometer (Figures S6 and S7). The DoE software MODDE Pro Version 12 (Sartorius Stedim, Göttingen, Germany) was used for parameter screening. We evaluated parameters possibly affecting sequence coverage, matched intensity, number of identified peptides as well as the average peptide length. A half fractional factorial design with three center points was selected, giving a 5+ resolution design with 19 runs in total (N1–N19). While all reaction mixtures had a pH value of approximately 2.5 (optimum value for pepsin digestion), the following input parameters were varied: total flow rate of the reaction mixture (1 to 5 μL·min^–1^), the distance between the sprayer and the MS inlet (3 to 7 cm), the concentrations of protein, protease, and buffer additives, and the nitrogen back pressure at the ESSI sprayer (60 to 120 psi). The autotune function of MODDE’s analysis wizard was used for model optimization, and nonsignificant factors were automatically removed by the algorithm. For a more detailed description, see the Supporting Information.

### ESSI Mass spectrometry

All experiments were carried out on an LTQ Orbitrap XL hybrid Ion Trap-Orbitrap mass spectrometer (ThermoFisher Scientific, San Jose, CA) equipped with an in-house built ESSI^29^ sprayer head (for details, see Figure S7). The following parameters were used for all MS experiments: positive ion mode, 3 kV spray voltage, 0 V capillary voltage, 200 °C capillary temperature, +55 V tube lens voltage, full MS range from *m*/*z* = 180 to 2000, 1000 ms max injection time, resolution was set to 60 000 at *m*/*z* = 200, 1 min total acquisition time per run. The obtained mass spectra were analyzed and annotated using Mmass V5.5.0 open source software.^30^ For details, see the Supporting Information.

## Results and Discussion

**Figure 1:**
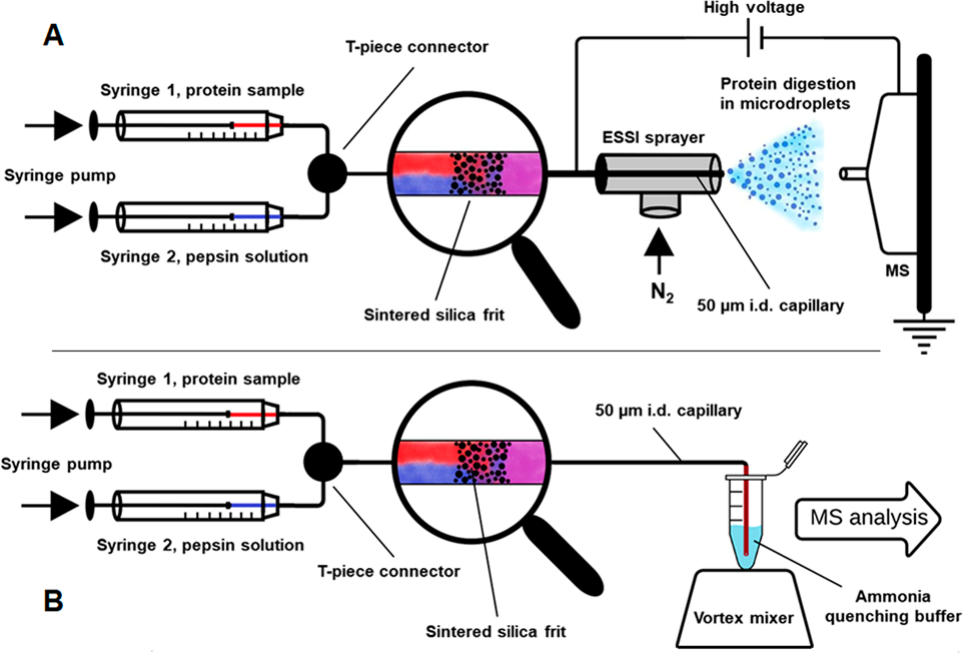
Schematic representations of the experimental setup: in-spray pepsin digestion utilizing online ESSI-MS (A) and the corresponding control experiment, in which ammonia was used to quench the protein/pepsin mixture at the exit of the capillary (B).

A simple experimental setup for in-spray protein digestion in microdroplets has been described previously.^24,26^ However, it has to be considered that the enzymatic degradation reaction immediately starts as soon as protein and protease solutions are mixed. Since premixing and filling into a single syringe is difficult to reproduce, we used two syringes to separately dispense the two reactants. The experimental setup depicted in Figure 1 allowed us (i) to reproducibly control online mixing of protease and protein solutions and (ii) to determine how much protein is being digested before the reaction mixture reaches the sprayer and microdroplets are formed.

### In-Spray Digestion of Cytochrome *c* Using Pepsin

**Figure 2:**
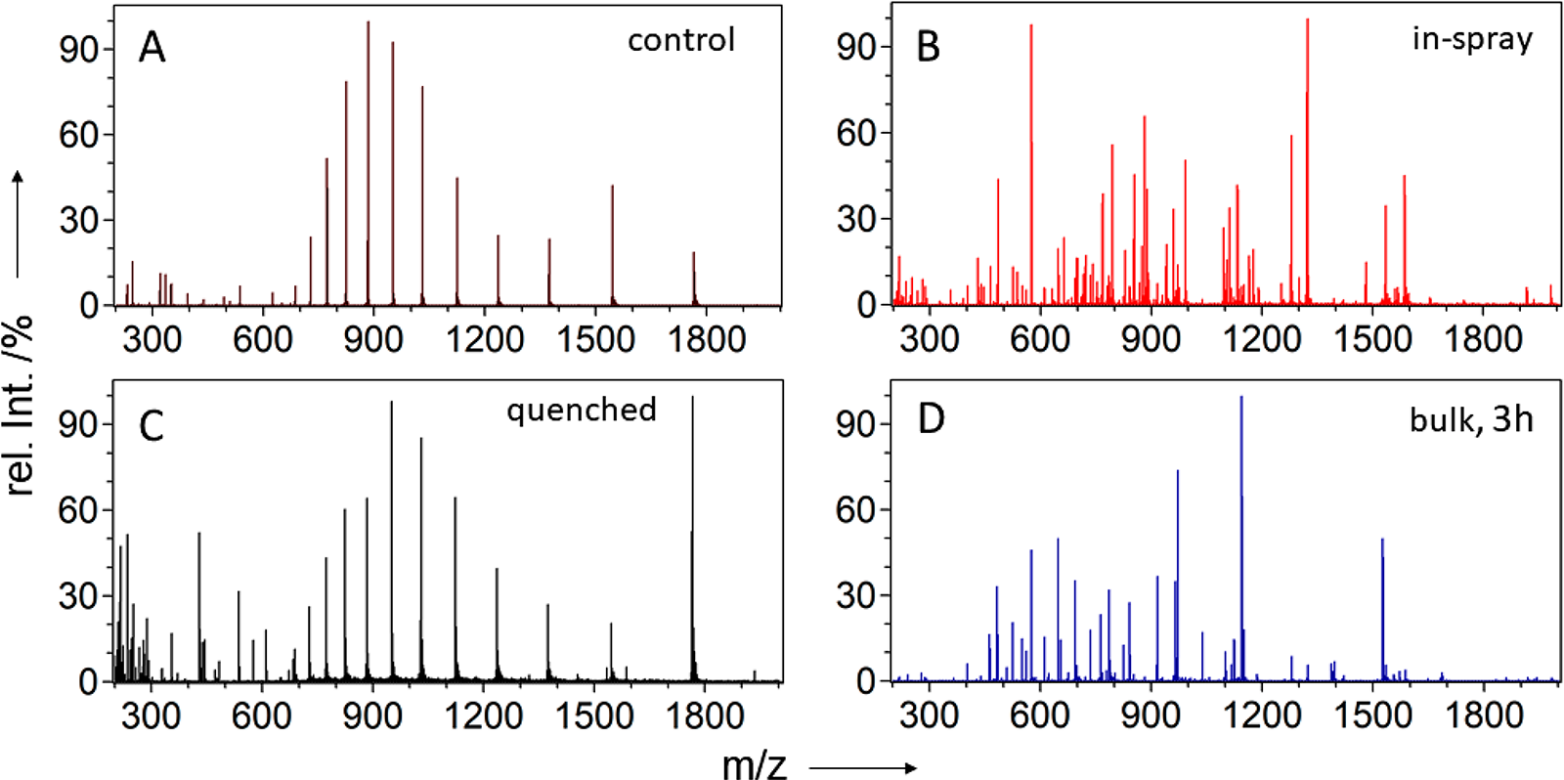
Positive ion-mode ESSI mass spectra of cytochrome *c*: mass spectrum of the intact protein dissolved in 4% formic acid (A), in-spray digestion of cytochrome *c* according to experiment 1A (B), mass spectrum of the reacidified, ammonia-quenched reaction mixture from the control experiment 1B (C), and mass spectrum of cytochrome *c* after 3 h of pepsin digestion in the bulk phase (D). For details, see the Experimental Section and the Supporting Information.

To exclude protein cleavage under harsh acidic conditions and in absence of protease, we performed simple preliminary experiments. Cytochrome *c* solutions were mixed 1:1 (v/v) with 8% formic acid or 20% acetic acid and sprayed for ESSI-MS analysis. The obtained mass spectra matched those from the intact protein; that is, no spontaneous cleavage was induced by ESSI at low pH values (Figures S1 and S2). For the in-spray digestion experiments, we used a dual syringe pump with two individual syringes for equal flow rates of protein solution *Cyt3* as well as protease solution *Pep3* (Figure 1A). After merging the two flows using a zero dead volume T-piece, a downstream sintered silica frit was used for thorough online mixing of the two solutions by disrupting the laminar flow within the narrow capillary.^31^ Finally, the mixture was sprayed toward the MS inlet using an in-lab built ESSI sprayer head, while the applied spray voltage was drawn from the mass spectrometer. Typical charge states of the intact protein could be observed when syringe 1 was filled with cytochrome *c* solution and syringe 2 with aqueous acetic or formic acid (Figure 2A). However, cytochrome *c* was readily digested when pepsin solution was dispensed with syringe 2. In this case, the obtained mass spectra showed numerous peptide signals, while signals corresponding to the intact protein could no longer be observed (Figure 2B; for peptide annotation, see Table S1). For comparison, an ESSI-MS analysis of cytochrome *c* after 3 h of bulk phase pepsin digestion was performed (Figure 2D). Due to a number of shorter peptides at lower charge states the bulk digest spectrum showed a slightly different set of signals (for experimental details, see the Supporting Information).

### Distinguishing Slow Digestion in Bulk from Ultrarapid Digestion in Microdroplets

A control experiment, schematically depicted in Figure 1B, was performed to precisely assess the individual contributions of bulk reaction and in-spray cleavage to the overall protein digestion. Therefore, the end of the outlet capillary was immersed in a 0.5% ammonia quenching solution (pH 11.4) throughout the experiment. An attached vortex mixer ensured that the protein/protease reaction mixture exiting the capillary was immediately quenched due to the irreversible inactivation of pepsin at basic pH values. After reacidification of the mixture, ESSI-MS analysis was performed. As can be seen in Figure 2C, the most abundant signals are typical charge states of intact cytochrome *c*. The absence of signals corresponding to enzymatic digestion products indicates that only a negligible amount of protein digestion occurs between mixing of protein and protease and pepsin inactivation by the quenching solution. Hence, we conclude that the comparably slow bulk reaction in the capillary does not contribute to the observed thorough protein digestion (e.g., Figure 2B), and virtually all protein cleavage must have occurred within the spray. We estimated the digestion time^24^ within the airborne microdroplets between sprayer and MS inlet to be below 1 ms by assuming a droplet speed^6,26^ of ∼80 m·s^–1^. In comparison to at least 1 h of incubation time recommended by standard protocols for digestion in bulk solution, we hypothesize a speed improvement by a factor greater than 10^6^.

### Experiment Optimization Using a DoE Approach

For efficient screening of experimental parameters, we performed different experimental runs according to a DoE design (for details, see the Experimental Section and the Supporting Information). The input variables were as follows: flow rate, distance between sprayer and MS inlet, concentration of protein and buffer additives, concentration of protease and buffer composition and N_2_ pressure. As output parameters, we defined the sequence coverage of the protein, the matched intensity, the number of identified peptides, as well as the average peptide length. After each run, the apparatus was flushed with 5 mM NH_4_HCO_3_ solution.

**Figure 3:**
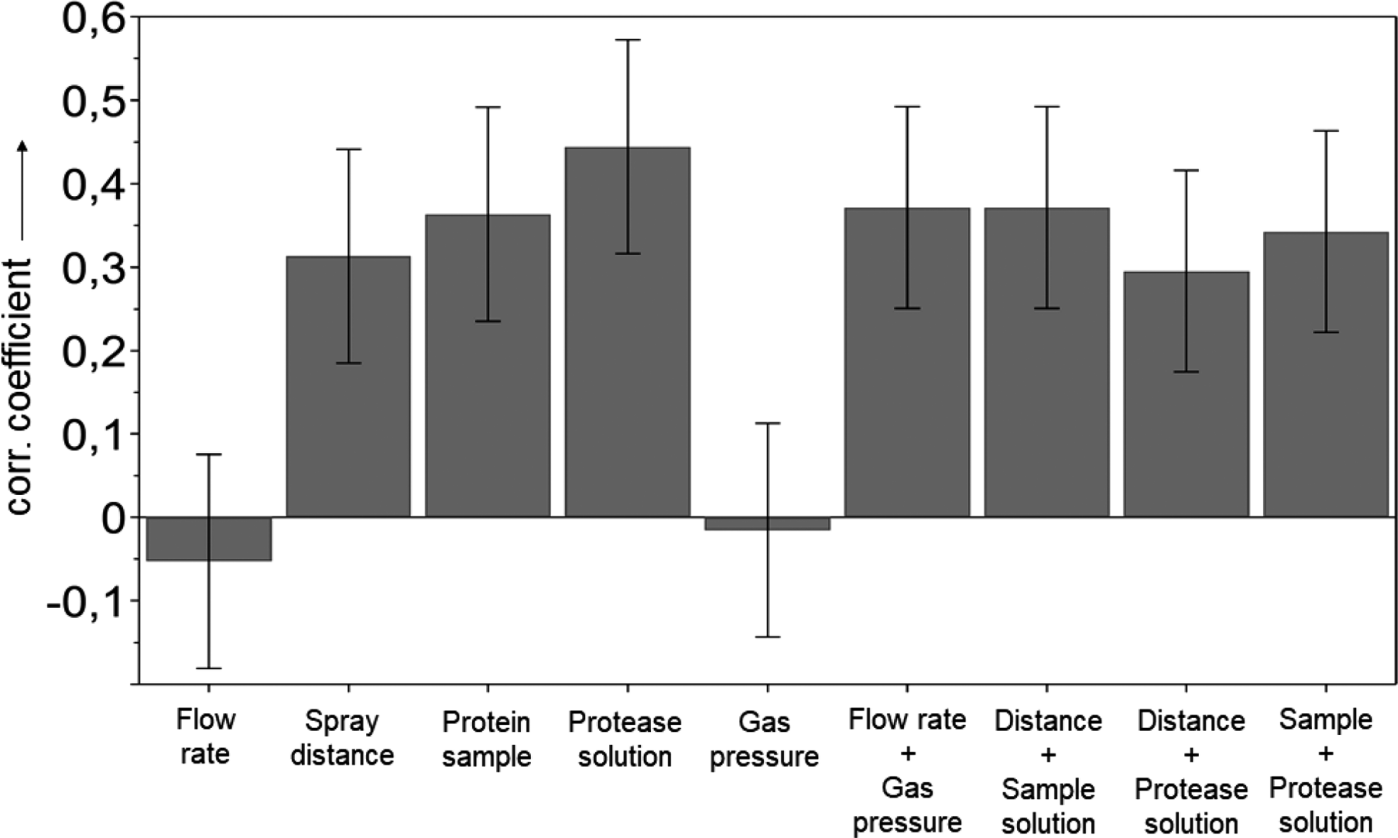
DoE screening results. Normalized coefficients plot for the output variable sequence coverage obtained by an analysis of 19 randomized DoE runs (*R*2 = 0.97, *N* = 19, DF = 9, 95% confidence). For further details, see the Experimental Section as well as the Supporting Information (Tables S3 and S4).

As can be seen in Figure 3, the DoE screening for the output variable sequence coverage identified seven factors which affected the total sequence coverage achieved by in-spray digestion of cytochrome *c*: (i) the distance between sprayer and MS inlet, (ii) the composition of the protein sample, (iii) the composition of protease solution, and the square test factors (iv) flow rate and gas pressure, (v) distance of the sprayer and protein sample, (vi) distance of the sprayer and protease solution, as well as (vii) protein sample and protease solution. However, positive correlation factors indicate positive contributions of the individual factor to the obtained sequence coverage in the DoE data set. The opposite is true for negative correlations. Hence, the use of solutions *Cyt3* and *Pep3* as well as greater sprayer distances increased the sequence coverage considerably, while flow rate and gas pressure had the least effect on the sequence coverage within the defined design space. The conditions for the best performing DoE run N16 (see also Table S3) were a total flow rate of 5 μL·min^–1^, 7 cm distance between sprayer and MS inlet, sample *Cyt3*, protease solution *Pep3*, and 120 psi nitrogen back pressure at the ESSI sprayer. This set of parameters resulted in a sequence coverage of 98.1% with 24.7% matched intensity and an average peptide length of 25 amino acids. A comparison of the worst in-spray experiment N2 (Figure S3) and N16 (Figure S4) clearly demonstrates that almost no cytochrome *c* was digested in run N2, despite the presence of protease at pH optimum for pepsin digestions. Hence, for efficient in-spray protein digestion, the experimental parameters have to be chosen carefully.

### In-Spray Digestion of Recombinant RocC Protein

**Figure 4:**
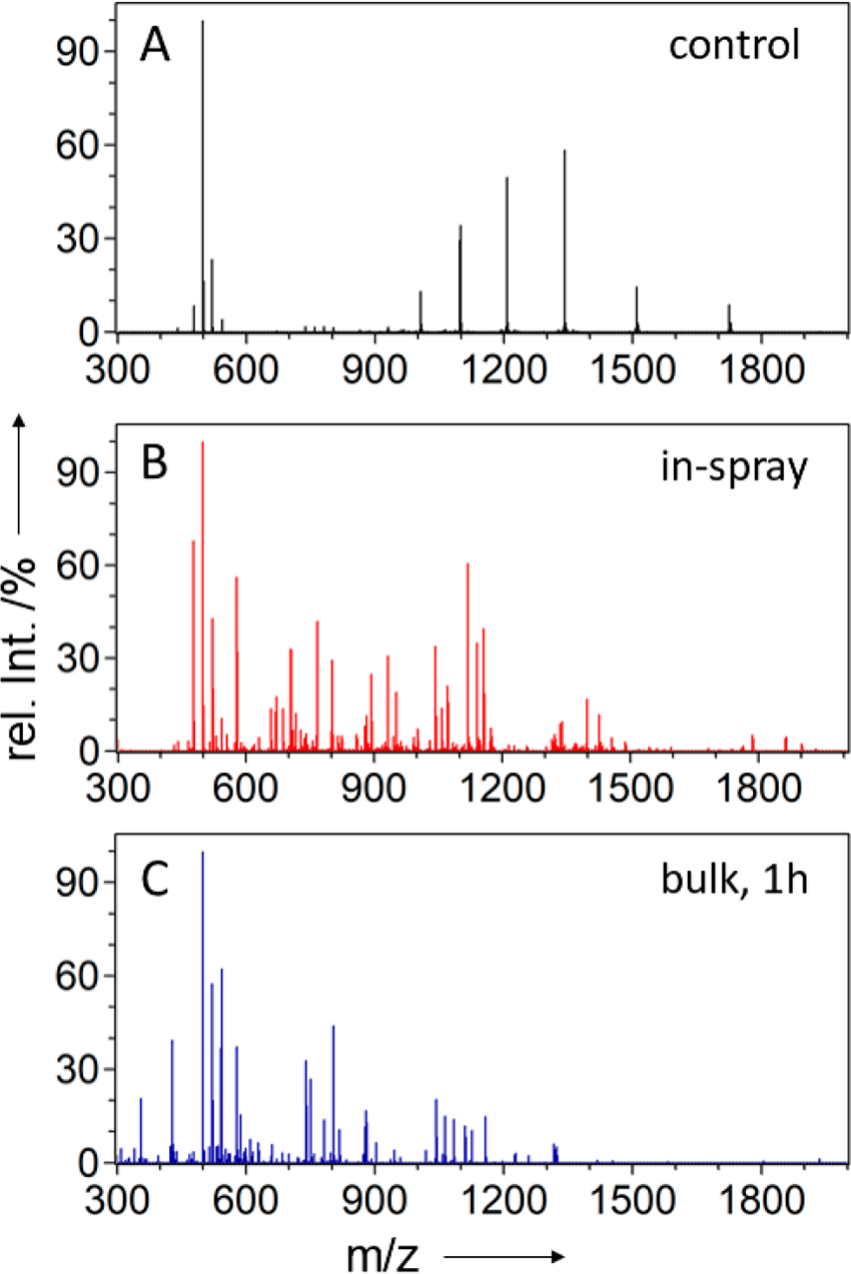
Positive ion-mode ESSI mass spectra of the recombinant RocC protein: mass spectrum of the intact protein in 4% formic acid (A), in-spray digestion of RocC according to experiment 1A (B), and mass spectrum of RocC after 1 h of pepsin digestion in the bulk phase (C). Signals around *m*/*z* = 500 were identified as artifacts from the protein purification and did not affect the MS analysis.

As can be seen in Figure 4, the developed method was successfully applied for the pepsin digestion of an authentic sample, namely, the in-house produced recombinant RNA chaperone RocC (residues 24–126). Figure 4A shows the charge states of the intact protein, while the spectrum depicted in Figure 4B was obtained when the protein was sprayed along with the pepsin solution *Pep3*. The spectrum depicted in Figure 4C was obtained by MS analysis after 1 h of in-bulk pepsin digestion (using *Pep3* at room temperature), followed by basic pepsin inactivation and reacidification prior to MS analysis.

The mass spectra from the in-spray digestion experiment (Figure 4B) showed more than 100 peptide signals, whereas the average peptide length was found to be 27 amino acids. Although a number of overlapping peptides were observed due to nonspecific cleavage by pepsin and potential miscleavages, 100% sequence coverage was achieved using optimized experimental settings (for details and peptide annotation, see Table S2 in the Supporting Information).

## Conclusion

Our experimental setup is characterized by a robust design relying on readily available and easily procurable materials. It allows for the fast screening of experimental conditions such as distance, flow rate, capillary temperature, spray voltage, etc. without compromising reproducibility and comparability between individual runs. Extremely accelerated peptic digestion, completed within milliseconds, was observed for commercially available as well as in-house produced proteins, displaying sequence coverage of 100% for RocC and 98.1% for cytochrome *c*. Our control experiments clearly showed that no spontaneous cleavage of the proteins was induced, even at harsh acidic conditions within the ESSI spray. Moreover, the quenching experiments conclusively showed that almost no protein is digested in the capillaries prior to microdroplet formation. The overwhelming proportion of protein is digested within the charged microdroplets at an exceptional speed. The vast number and abundance as well as average length of the observed peptides qualifies this technique to be a viable tool for the simplification of future protein HDX experiments. We anticipate that ultrarapid protein digestions will facilitate extremely fast HDX experiments and thereby increase throughput as well as reduce H/D back-exchange significantly.

## Supporting Information Available

Data processing; ESSI mass spectra of cytochrome *c* in acidic aqueous solutions (Figures S1 and S2); annotation of peptides obtained by in-spray digestion of cytochrome *c* (Table S1) and RocC_24–126_ (Table S2); design of experiment screening (Tables S3–S5 and Figures S3–S5); experimental setup (Figures S6 and S7); control experiments (bulk digestion and quenching experiment (PDF)
