# Supplementary material for: Microdroplet Mass Spectrometry Enables Extremely Accelerated Pepsin Digestion of Proteins
Source: J Am Soc Mass Spectrom. 2021 Jun 8;32(7):1841–5. doi: 10.1021/jasms.1c00126 (PMC8267842; doi:10.1021/jasms.1c00126)
Supplement: Supplementary file 1 — js1c00126_si_001.pdf [file js1c00126_si_001.pdf]

Microdroplet Mass Spectrometry Enables Extremely Accelerated Pepsin Digestion of Proteins

Tobias Rainer, Reiner Eidelpes, Martin Tollinger and Thomas Müller*

Institute of Organic Chemistry and Center for Molecular Biosciences (CMBI), Leopold-Franzens University Innsbruck, 6020 Innsbruck, Austria

*E-mail: thomas.mueller@uibk.ac.at

Supporting Information

Data processing

The obtained full MS raw data (acquisition time 1 min) were averaged, exported as .raw files using Xcalibur Qual Browser 4.1 software (Thermo Scientific, San Jose, CA), and converted to mzML format using the open source software MSconvert V3.0.[1] The open source tool mMass V5.5.0[2] was used for peak picking, in silico protein digestion and peptide identification. The mMass sequence function was used to calculate all possible peptide signals up to charge state 8+ (allowing variable methionine oxidation). The computed peptides were matched with the spectra and annotated (mass tolerance ± 5 ppm). All fitted signals were checked manually for isotope, charge and m/z accuracy. Non-matching annotations were deleted.

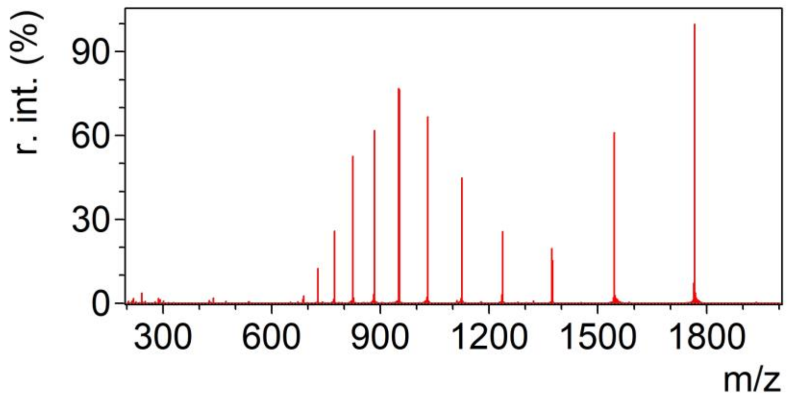

Figure S1. ESSI-Spectrum of cytochrome c sample *Cyt3* mixed 1:1 (v/v) with 20% acetic acid.

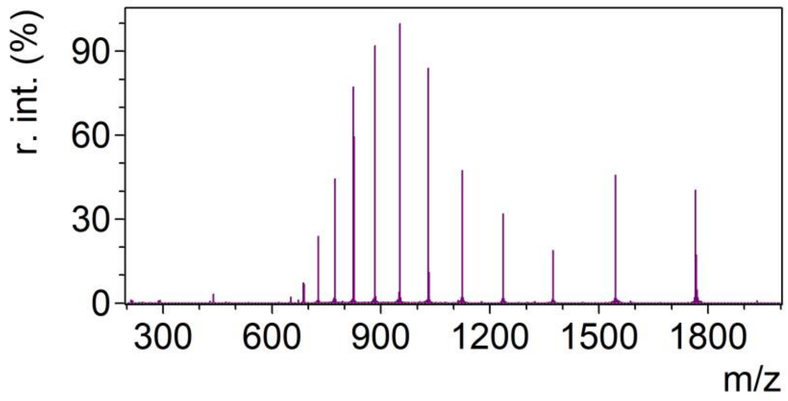

Figure S2. ESSI-Spectrum of cytochrome c sample *Cyt3* mixed 1:1 (v/v) with 8% formic acid.

Annotation of peptides obtained by in-spray digestion of cytochrome c

In-spray digestion of cytochrome c using ESSI-MS[3]: experimental setup as depicted in Figures 1A, S6 and S7, DoE Run N16 showed the best results in terms of sequence coverage at the following settings: flow rate 5 µL·min^{-1}, 7 cm distance between sprayer and MS inlet, sample Cyt3, protease solution Pep3, 120 psi nitrogen back pressure. The obtained sequence coverage was 98.1 %, matched intensity was found to be 24.1 %.

Cytochrome c from horse heart: Sequence length 105, average mass 11.880 kDa, single letter code sequence: MGDVEKGKKI FVQKCAQCHT VEKGGKHKTG PNLHGLFGRK TGQAPGFTYT DANKNKGITW KEETLMEYLE NPKKYIPGTK MIFAGIKKKT EREDLIAYLK KATNE

Table S1. Annotated peptides found for in-spray digestion of cytochrome c in DoE run N16. Mass tolerance was set to ±5 ppm.

m/z meas.	z	Annotation
402.2345	2	[3-9] g.DVEKGKK.i
463.2682	3	[69-80] y.LENPKKYIPGTK.m
463.2682	3	[84-95] f.AGIKKKTEREDL.i
483.7668	2	[98-105] a.YLKKATNE.
509.7753	2	[38-47] f.GRKTGQAPGF.t
509.7753	2	[37-46] l.FGRKTGQAPG.f
524.6426	3	[84-97] f.AGIKKKTEREDLIA.y
549.9862	3	[82-95] m.IFAGIKKKTEREDL.i
549.9862	3	[83-96] i.FAGIKKKTEREDLI.a
561.3044	3	[68-81] e.YLENPKKYIPGTKM.i
575.8275	2	[96-105] l.IAYLKKATNE.
602.2832	2	[14-24] q.KCAQCHTVEKG.g
611.3598	3	[82-97] m.IFAGIKKKTEREDLIA.y
630.6102	4	[84-105] f.AGIKKKTEREDLIAYLKKATNE.
648.0197	3	[68-83] e.YLENPKKYIPGTKMIF.a
662.9703	5	[68-95] e.YLENPKKYIPGTKMIFAGIKKKTEREDL.i
694.3991	2	[69-80] y.LENPKKYIPGTK.m
694.3991	2	[84-95] f.AGIKKKTEREDL.i
695.6486	4	[82-105] m.IFAGIKKKTEREDLIAYLKKATNE.

699.7959	5	[70-99] l.ENPKKYIPGTKMIFAGIKKKTEREDLIAYL.k
699.7959	5	[69-98] y.LENPKKYIPGTKMIFAGIKKKTEREDLIAY.l
699.7959	5	[68-97] e.YLENPKKYIPGTKMIFAGIKKKTEREDLIA.y
714.9864	5	[65-94] t.LMEYLENPKKYIPGTKMIFAGIKKKTERED.l
714.9864	5	[66-95] l.MEYLENPKKYIPGTKMIFAGIKKKTEREDL.i
734.7158	3	[66-83] l.MEYLENPKKYIPGTKMIF.a
741.2498	6	[67-104] m.EYLENPKKYIPGTKMIFAGIKKKTEREDLIAYLKKATN.e
741.2498	6	[68-105] e.YLENPKKYIPGTKMIFAGIKKKTEREDLIAYLKKATNE.
751.8097	5	[66-97] l.MEYLENPKKYIPGTKMIFAGIKKKTEREDLIA.y
778.65	4	[38-65] f.GRKTGQAPGFTYTDANKNKGITWKEETL.m
784.5966	6	[66-105] l.MEYLENPKKYIPGTKMIFAGIKKKTEREDLIAYLKKATNE.
786.4593	2	[84-97] f.AGIKKKTEREDLIA.y
824.4746	2	[83-96] i.FAGIKKKTEREDLI.a
828.4612	4	[68-95] e.YLENPKKYIPGTKMIFAGIKKKTEREDL.i
843.6737	4	[38-67] f.GRKTGQAPGFTYTDANKNKGITWKEETLME.y
860.7349	4	[68-96] e.YLENPKKYIPGTKMIFAGIKKKTEREDLI.a [1xOxidation]
874.4914	4	[68-97] e.YLENPKKYIPGTKMIFAGIKKKTEREDLIA.y
874.4914	4	[69-98] y.LENPKKYIPGTKMIFAGIKKKTEREDLIAY.l
874.4914	4	[70-99] l.ENPKKYIPGTKMIFAGIKKKTEREDLIAYL.k
889.297	5	[67-104] m.EYLENPKKYIPGTKMIFAGIKKKTEREDLIAYLKKATN.e
889.297	5	[68-105] e.YLENPKKYIPGTKMIFAGIKKKTEREDLIAYLKKATNE.
893.4803	4	[65-94] t.LMEYLENPKKYIPGTKMIFAGIKKKTERED.l
893.4803	4	[66-95] l.MEYLENPKKYIPGTKMIFAGIKKKTEREDL.i
927.197	3	[82-105] m.IFAGIKKKTEREDLIAYLKKATNE.
939.5118	4	[66-97] l.MEYLENPKKYIPGTKMIFAGIKKKTEREDLIA.y
941.3125	5	[66-105] l.MEYLENPKKYIPGTKMIFAGIKKKTEREDLIAYLKKATNE.
966.5257	1	[98-105] a.YLKKATNE.
971.5269	2	[68-83] e.YLENPKKYIPGTKMIF.a
980.5207	4	[3-38] g.DVEKGKKIFVQKCAQCHTVEKGGKHKTGPNLHGLFG.r
1037.87	3	[38-65] f.GRKTGQAPGFTYTDANKNKGITWKEETL.m
1101.5704	2	[66-83] l.MEYLENPKKYIPGTKMIF.a
1104.2797	3	[68-95] e.YLENPKKYIPGTKMIFAGIKKKTEREDL.i
1111.3697	4	[67-104] m.EYLENPKKYIPGTKMIFAGIKKKTEREDLIAYLKKATN.e
1111.3697	4	[68-105] e.YLENPKKYIPGTKMIFAGIKKKTEREDLIAYLKKATNE.
1143.5936	3	[35-65] h.GLFGRKTGQAPGFTYTDANKNKGITWKEETL.m
1150.6476	1	[96-105] l.IAYLKKATNE.
1165.6556	3	[69-98] y.LENPKKYIPGTKMIFAGIKKKTEREDLIAY.l
1165.6556	3	[68-97] e.YLENPKKYIPGTKMIFAGIKKKTEREDLIA.y
1165.6556	3	[70-99] l.ENPKKYIPGTKMIFAGIKKKTEREDLIAYL.k
1176.3945	4	[66-105] l.MEYLENPKKYIPGTKMIFAGIKKKTEREDLIAYLKKATNE.
1252.3497	3	[66-97] l.MEYLENPKKYIPGTKMIFAGIKKKTEREDLIA.y
1481.4962	3	[67-104] m.EYLENPKKYIPGTKMIFAGIKKKTEREDLIAYLKKATN.e
1481.4962	3	[68-105] e.YLENPKKYIPGTKMIFAGIKKKTEREDLIAYLKKATNE.
1568.1904	3	[66-105] l.MEYLENPKKYIPGTKMIFAGIKKKTEREDLIAYLKKATNE.

Annotation of peptides obtained by in-spray digestion of recombinant RocC$_{24\text{-}126}$

In-spray digestion of recombinant RocC$_{24\text{-}126}$ using ESSI-MS^3: experimental setup as depicted in Figures 1A, S6 and S7, flow rate was 1 µL·min^{-1}, 5 cm distance between sprayer and MS inlet, sample *RocC1* (approx. 0.26 mg·mL^{-1} in 5 mM NH_4HCO_3), protease solution Pep3, 70 psi nitrogen back pressure. Obtained sequence coverage was 100 %, matched intensity was found to be 60.3 %.

RocC$_{24\text{-}126}$ (in-house, recombinant with protease tag GPLGS): Sequence length 108, 12.069 kDa average mass, single letter code: GPLGSARSDA LLWLAANFPE AFDNSLRIRP LKIGIMSDIL QHAEKAEQVG VSKSKLREAV VLFTRRLDYL ACLKAREVRI DLHGNPVAEV TEEEAENASM KIKKRVEK

Table S2. Annotated peptides found for the in-spray digestion of RocC$_{24\text{-}126}$. Mass tolerance was set to ±5 ppm.

m/z meas.	z	Annotation
399.5923	3	[27-36] l.RIRPLKIGIM.s
431.2644	1	[11-13] a.LLW.l
431.2644	1	[12-14] l.LWL.a
437.287	3	[26-36] s.LRIRPLKIGIM.s
464.5047	4	[63-77] l.FTRRLDYLACLKARE.v
466.9457	3	[27-38] l.RIRPLKIGIMSD.i
468.7663	2	[64-70] f.TRRLDYL.a
504.6402	3	[27-39] l.RIRPLKIGIMSDI.l
504.6402	3	[26-38] s.LRIRPLKIGIMSD.i
522.277	2	[1-11] .GPLGSARSDAL.l
542.3001	2	[62-69] v.LFTRRLDY.l
542.3001	2	[63-70] l.FTRRLDYL.a
555.5472	4	[90-108] e.VTEEEAENASMKIKKRVEK.
563.3188	4	[39-58] d.ILQHAEKAEQVGVSKSKLRE.a
574.0513	7	[26-61] s.LRIRPLKIGIMSDILQHAEKAEQVGVSKSKLREAVV.l
574.0513	7	[27-62] l.RIRPLKIGIMSDILQHAEKAEQVGVSKSKLREAVVL.f
578.8189	2	[1-12] .GPLGSARSDALL.w
585.5032	5	[83-108] l.HGNPVAEVTEEEAENASMKIKKRVEK.
587.4816	6	[78-108] e.VRIDLHGNPVAEVTEEEAENASMKIKKRVEK.
598.8853	2	[27-36] l.RIRPLKIGIM.s
601.9979	3	[74-89] l.KAREVRIDLHGNPVAE.v
612.331	2	[64-73] f.TRRLDYLACL.k
615.3817	1	[78-82] e.VRIDL.h
619.0037	3	[63-77] l.FTRRLDYLACLKARE.v
629.3229	2	[63-72] l.FTRRLDYLAC.l
631.9421	5	[63-89] l.FTRRLDYLACLKAREVRIDLHGNPVAE.v
639.692	3	[73-89] c.LKAREVRIDLHGNPVAE.v
658.8831	4	[39-62] d.ILQHAEKAEQVGVSKSKLREAVVL.f
660.3564	2	[78-89] e.VRIDLHGNPVAE.v
660.3564	2	[79-90] v.RIDLHGNPVAEV.t
660.3564	2	[77-88] r.EVRIDLHGNPVA.e
668.1937	6	[74-108] l.KAREVRIDLHGNPVAEVTEEEAENASMKIKKRVEK.
669.5581	6	[26-61] s.LRIRPLKIGIMSDILQHAEKAEQVGVSKSKLREAVV.l
669.5581	6	[27-62] l.RIRPLKIGIMSDILQHAEKAEQVGVSKSKLREAVVL.f
670.4848	8	[62-107] v.LFTRRLDYLACLKAREVRIDLHGNPVAEVTEEEAENASMKIKKRVE.k [1xOxidation]
685.8648	2	[62-72] v.LFTRRLDYLAC.l
685.8648	2	[63-73] l.FTRRLDYLACL.k
695.6495	4	[39-63] d.ILQHAEKAEQVGVSKSKLREAVVLF.t

697.7075	3	[71-89] l.ACLKAREVRIDLHGNPVAE.v
699.9144	2	[27-38] l.RIRPLKIGIMSD.i
704.7758	5	[78-108] e.VRIDLHGNPVAEVTEEEAENASMKIKKRVEK.
704.7758	5	[75-105] k.AREVRIDLHGNPVAEVTEEEAENASMKIKKR.v
709.3965	4	[37-62] m.SDILQHAEKAEQVGVSKSKLREAVVL.f
709.3965	4	[29-54] i.RPLKIGIMSDILQHAEKAEQVGVSKS.k
716.0476	6	[71-108] l.ACLKAREVRIDLHGNPVAEVTEEEAENASMKIKKRVEK.
728.3989	2	[1-14] .GPLGSARSDALLWL.a
731.6265	4	[83-108] l.HGNPVAEVTEEEAENASMKIKKRVEK.
740.3927	3	[90-108] e.VTEEEAENASMKIKKRVEK.
746.1635	4	[37-63] m.SDILQHAEKAEQVGVSKSKLREAVVLF.t
754.7302	3	[74-93] l.KAREVRIDLHGNPVAEVTEE.e
765.9813	7	[63-108] l.FTRRLDYLACLKAREVRIDLHGNPVAEVTEEEAENASMKIKKRVEK.
789.6762	4	[63-89] l.FTRRLDYLACLKAREVRIDLHGNPVAE.v
799.1556	7	[12-61] l.LWLAANFPEAFDNSLRIRPLKIGIMSDILQHAEKAEQVGVSKSKLREAVV.l
799.1556	7	[13-62] l.WLAANFPEAFDNSLRIRPLKIGIMSDILQHAEKAEQVGVSKSKLREAVVL.f
801.6303	5	[74-108] l.KAREVRIDLHGNPVAEVTEEEAENASMKIKKRVEK.
803.2675	5	[27-62] l.RIRPLKIGIMSDILQHAEKAEQVGVSKSKLREAVVL.f
803.2675	5	[26-61] s.LRIRPLKIGIMSDILQHAEKAEQVGVSKSKLREAVV.l
815.3086	7	[12-62] l.LWLAANFPEAFDNSLRIRPLKIGIMSDILQHAEKAEQVGVSKSKLREAVVL.f
815.3086	7	[11-61] a.LLWLAANFPEAFDNSLRIRPLKIGIMSDILQHAEKAEQVGVSKSKLREAVV.l
824.2471	5	[73-108] c.LKAREVRIDLHGNPVAEVTEEEAENASMKIKKRVEK.
825.8841	5	[26-62] s.LRIRPLKIGIMSDILQHAEKAEQVGVSKSKLREAVVL.f
859.0562	5	[71-108] l.ACLKAREVRIDLHGNPVAEVTEEEAENASMKIKKRVEK.
878.174	3	[39-62] d.ILQHAEKAEQVGVSKSKLREAVVL.f
880.7174	4	[78-108] e.VRIDLHGNPVAEVTEEEAENASMKIKKRVEK.
880.7174	4	[75-105] k.AREVRIDLHGNPVAEVTEEEAENASMKIKKR.v
882.3185	6	[14-61] w.LAANFPEAFDNSLRIRPLKIGIMSDILQHAEKAEQVGVSKSKLREAVV.l
882.3185	6	[15-62] l.AANFPEAFDNSLRIRPLKIGIMSDILQHAEKAEQVGVSKSKLREAVVL.f
892.4681	3	[15-38] l.AANFPEAFDNSLRIRPLKIGIMSD.i
902.4922	2	[74-89] l.KAREVRIDLHGNPVAE.v
908.5202	3	[49-72] q.VGVSKSKLREAVVLFTRRLDYLAC.l
916.5159	3	[38-62] s.DILQHAEKAEQVGVSKSKLREAVVL.f
916.5159	3	[29-53] i.RPLKIGIMSDILQHAEKAEQVGVSK.s
918.5185	5	[22-62] a.FDNSLRIRPLKIGIMSDILQHAEKAEQVGVSKSKLREAVVL.f
918.5185	5	[23-63] f.DNSLRIRPLKIGIMSDILQHAEKAEQVGVSKSKLREAVVLF.t
924.8355	3	[13-36] l.WLAANFPEAFDNSLRIRPLKIGIM.s
927.1973	3	[39-63] d.ILQHAEKAEQVGVSKSKLREAVVLF.t
928.0018	2	[63-77] l.FTRRLDYLACLKARE.v
932.1803	6	[12-61] l.LWLAANFPEAFDNSLRIRPLKIGIMSDILQHAEKAEQVGVSKSKLREAVV.l
932.1803	6	[13-62] l.WLAANFPEAFDNSLRIRPLKIGIMSDILQHAEKAEQVGVSKSKLREAVVL.f
945.5266	3	[37-62] m.SDILQHAEKAEQVGVSKSKLREAVVL.f
945.5266	3	[29-54] i.RPLKIGIMSDILQHAEKAEQVGVSKS.k
951.0258	6	[11-61] a.LLWLAANFPEAFDNSLRIRPLKIGIMSDILQHAEKAEQVGVSKSKLREAVV.l
951.0258	6	[12-62] l.LWLAANFPEAFDNSLRIRPLKIGIMSDILQHAEKAEQVGVSKSKLREAVVL.f
953.8486	3	[13-37] l.WLAANFPEAFDNSLRIRPLKIGIMS.d
962.5306	3	[12-36] l.LWLAANFPEAFDNSLRIRPLKIGIM.s
988.0431	2	[22-38] a.FDNSLRIRPLKIGIMSD.i
992.1894	3	[13-38] l.WLAANFPEAFDNSLRIRPLKIGIMSD.i
994.55	3	[37-63] m.SDILQHAEKAEQVGVSKSKLREAVVLF.t
1001.7867	4	[74-108] l.KAREVRIDLHGNPVAEVTEEEAENASMKIKKRVEK.
1003.8326	4	[26-61] s.LRIRPLKIGIMSDILQHAEKAEQVGVSKSKLREAVV.l
1003.8326	4	[27-62] l.RIRPLKIGIMSDILQHAEKAEQVGVSKSKLREAVVL.f
1018.4966	1	[13-21] l.WLAANFPEA.f
1029.8843	3	[12-38] l.LWLAANFPEAFDNSLRIRPLKIGIMSD.i
1032.1044	4	[26-62] s.LRIRPLKIGIMSDILQHAEKAEQVGVSKSKLREAVVL.f

1041.9611	5	[13-58] l.WLAANFPEAFDNSLRIRPLKIGIMSDILQHAEKAEQVGVSKSKLRE.a
1043.5452	1	[1-11] .GPLGSARSDAL.l
1052.5654	3	[63-89] l.FTRRLDYLACLKAREVRIDLHGNPVAE.v
1058.5824	5	[15-62] l.AANFPEAFDNSLRIRPLKIGIMSDILQHAEKAEQVGVSKSKLREAVVL.f
1058.5824	5	[14-61] w.LAANFPEAFDNSLRIRPLKIGIMSDILQHAEKAEQVGVSKSKLREAVV.l
1064.5872	5	[11-57] a.LLWLAANFPEAFDNSLRIRPLKIGIMSDILQHAEKAEQVGVSKSKLR.e [1xOxidation]
1064.5872	5	[12-58] l.LWLAANFPEAFDNSLRIRPLKIGIMSDILQHAEKAEQVGVSKSKLRE.a
1071.9703	5	[63-108] l.FTRRLDYLACLKAREVRIDLHGNPVAEVTEEEAENASMKIKKRVEK.
1073.5692	4	[71-108] l.ACLKAREVRIDLHGNPVAEVTEEEAENASMKIKKRVEK.
1083.5921	1	[62-69] v.LFTRRLDY.l
1083.5921	1	[63-70] l.FTRRLDYL.a
1118.4151	5	[12-61] l.LWLAANFPEAFDNSLRIRPLKIGIMSDILQHAEKAEQVGVSKSKLREAVV.l
1118.4151	5	[13-62] l.WLAANFPEAFDNSLRIRPLKIGIMSDILQHAEKAEQVGVSKSKLREAVVL.f
1131.5825	1	[12-21] l.LWLAANFPEA.f
1141.0311	5	[11-61] a.LLWLAANFPEAFDNSLRIRPLKIGIMSDILQHAEKAEQVGVSKSKLREAVV.l
1141.0311	5	[12-62] l.LWLAANFPEAFDNSLRIRPLKIGIMSDILQHAEKAEQVGVSKSKLREAVVL.f
1156.6291	1	[1-12] .GPLGSARSDALL.w
1165.5665	1	[13-22] l.WLAANFPEAF.d
1173.9557	3	[78-108] e.VRIDLHGNPVAEVTEEEAENASMKIKKRVEK.
1188.1325	2	[73-93] c.LKAREVRIDLHGNPVAEVTEE.e
1205.6342	4	[1-44] .GPLGSARSDALLWLAANFPEAFDNSLRIRPLKIGIMSDILQHAE.k [1xOxidation]
1223.655	1	[64-73] f.TRRLDYLACL.k
1257.6382	1	[63-72] l.FTRRLDYLAC.l
1295.5874	1	[14-25] w.LAANFPEAFDNS.l
1295.5874	1	[15-26] l.AANFPEAFDNSL.r
1316.7594	2	[39-62] d.ILQHAEKAEQVGVSKSKLREAVVL.f
1319.7061	1	[77-88] r.EVRIDLHGNPVA.e
1319.7061	1	[78-89] e.VRIDLHGNPVAE.v
1319.7061	1	[79-90] v.RIDLHGNPVAEV.t
1322.979	4	[14-61] w.LAANFPEAFDNSLRIRPLKIGIMSDILQHAEKAEQVGVSKSKLREAVV.l
1322.979	4	[15-62] l.AANFPEAFDNSLRIRPLKIGIMSDILQHAEKAEQVGVSKSKLREAVVL.f
1335.3819	3	[74-108] l.KAREVRIDLHGNPVAEVTEEEAENASMKIKKRVEK.
1338.112	3	[26-61] s.LRIRPLKIGIMSDILQHAEKAEQVGVSKSKLREAVV.l
1338.112	3	[27-62] l.RIRPLKIGIMSDILQHAEKAEQVGVSKSKLREAVVL.f
1339.7417	4	[33-79] k.IGIMSDILQHAEKAEQVGVSKSKLREAVVLFTRRLDYLACLKAREVR.i [1xOxidation]
1370.7241	1	[62-72] v.LFTRRLDYLAC.l
1370.7241	1	[63-73] l.FTRRLDYLACL.k
1373.0769	3	[36-71] i.MSDILQHAEKAEQVGVSKSKLREAVVLFTRRLDYLA.c [1xOxidation]
1373.0769	3	[73-108] c.LKAREVRIDLHGNPVAEVTEEEAENASMKIKKRVEK.
1386.7541	2	[13-36] l.WLAANFPEAFDNSLRIRPLKIGIM.s
1390.2917	2	[39-63] d.ILQHAEKAEQVGVSKSKLREAVVLF.t
1397.7701	4	[12-61] l.LWLAANFPEAFDNSLRIRPLKIGIMSDILQHAEKAEQVGVSKSKLREAVV.l
1397.7701	4	[13-62] l.WLAANFPEAFDNSLRIRPLKIGIMSDILQHAEKAEQVGVSKSKLREAVVL.f
1417.7884	2	[37-62] m.SDILQHAEKAEQVGVSKSKLREAVVL.f
1417.7884	2	[29-54] i.RPLKIGIMSDILQHAEKAEQVGVSKS.k
1426.0414	4	[11-61] a.LLWLAANFPEAFDNSLRIRPLKIGIMSDILQHAEKAEQVGVSKSKLREAVV.l
1426.0414	4	[12-62] l.LWLAANFPEAFDNSLRIRPLKIGIMSDILQHAEKAEQVGVSKSKLREAVVL.f
1431.0911	3	[71-108] l.ACLKAREVRIDLHGNPVAEVTEEEAENASMKIKKRVEK.
1443.297	2	[12-36] l.LWLAANFPEAFDNSLRIRPLKIGIM.s
1455.7971	1	[1-14] .GPLGSARSDALLWL.a
1481.669	1	[13-25] l.WLAANFPEAFDNS.l
1487.7849	2	[13-38] l.WLAANFPEAFDNSLRIRPLKIGIMSD.i
1544.3245	2	[12-38] l.LWLAANFPEAFDNSLRIRPLKIGIMSD.i
1544.3245	2	[13-39] l.WLAANFPEAFDNSLRIRPLKIGIMSDI.l

1594.754	1	[12-25] l.LWLAANFPEAFDNS.l
1594.754	1	[13-26] l.WLAANFPEAFDNSL.r
1707.8404	1	[11-25] a.LLWLAANFPEAFDNS.l
1707.8404	1	[12-26] l.LWLAANFPEAFDNSL.r

Design of Experiment (DoE) screening

For the DoE screening model, the input parameters were set as flow rate (1, 2.5, 5 µl·min^{-1}), distance (3, 5, 7 cm), sample solution (*Cyt1, Cyt2, Cyt3*), protease solution (*Pep1, Pep2, Pep3),* N2 pressure (60, 90, 120 psi). Output parameters were defined: sequence coverage, matched intensity, number of identified peptides, average peptide length. A half fractional factorial interaction model with 3 center points was used, giving a 5+ resolution design with 19 runs in total.

Table S3. Experimental parameters and results of DoE runs.

Exp Name	Run Order	Flow rate /µl·min^{-1}	Distance /cm	Sample solution	Protease solution	Gas pressure /psi	Sequence coverage /%	Matched intensity /%	Number of peptides detected	Average peptide length
**N1**	3	1	3	Cyt1	Pep1	120	39	11.2	33	29
**N2**	15	5	3	Cyt1	Pep1	60	39	1.2	9	36
**N3**	11	1	7	Cyt1	Pep1	60	39	10.8	34	29
**N4**	1	5	7	Cyt1	Pep1	120	39	7.2	24	29
**N5**	8	1	3	Cyt3	Pep1	60	39	6.6	32	28
**N6**	10	5	3	Cyt3	Pep1	120	39	4	17	32
**N7**	2	1	7	Cyt3	Pep1	120	39	12	32	30
**N8**	19	5	7	Cyt3	Pep1	60	39	3.2	15	33
**N9**	16	1	3	Cyt1	Pep3	60	51.4	4.7	27	33
**N10**	14	5	3	Cyt1	Pep3	120	39	8.8	27	29
**N11**	18	1	7	Cyt1	Pep3	120	39	23.7	40	29
**N12**	17	5	7	Cyt1	Pep3	60	39	8.7	28	31
**N13**	12	1	3	Cyt3	Pep3	120	45.7	17.5	53	27
**N14**	13	5	3	Cyt3	Pep3	60	40	5.3	35	30
**N15**	4	1	7	Cyt3	Pep3	60	96.2	13.2	50	27
**N16**	5	5	7	Cyt3	Pep3	120	98.1	24.7	65	25
**N17**	9	2.5	5	Cyt2	Pep2	90	39	11.9	25	31
**N18**	6	2.5	5	Cyt2	Pep2	90	42.9	11.1	25	28
**N19**	7	2.5	5	Cyt2	Pep2	90	43.8	7.9	21	28

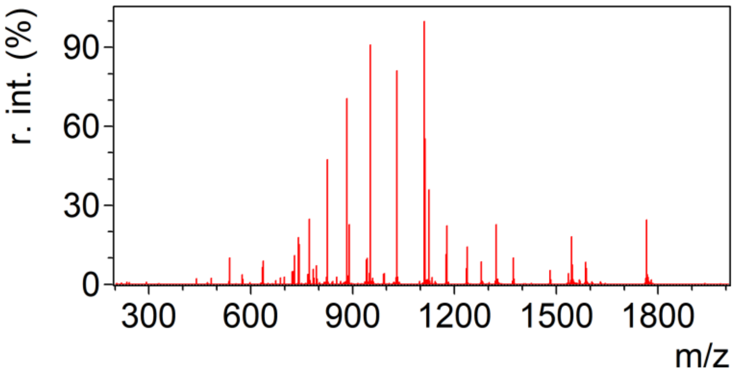

Figure S3. ESSI-MS spectrum of the worst performing DoE run (N2) of in-spray digestion of cytochrome c. Experimental setup as depicted in Figure 1A, flow rate was 5 µL·min^{-1}, 3 cm distance between sprayer and MS inlet, sample *Cyt1*, protease solution *Pep1*, 60 psi nitrogen back pressure.

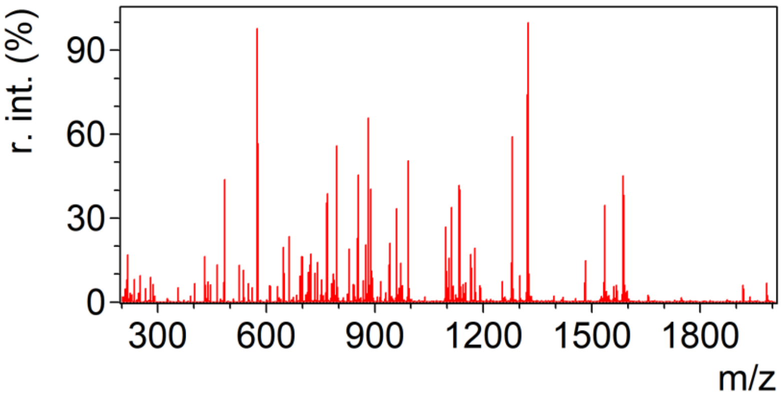

Figure S4. ESSI-MS spectrum of the best performing DoE run (N16) of in-spray digestion of cytochrome c. Experimental setup as depicted in Figure 1A flow rate was 5 µL·min^{-1}, 7 cm distance between sprayer and MS inlet, sample *Cyt3*, protease solution *Pep3*, 120 psi nitrogen back pressure.

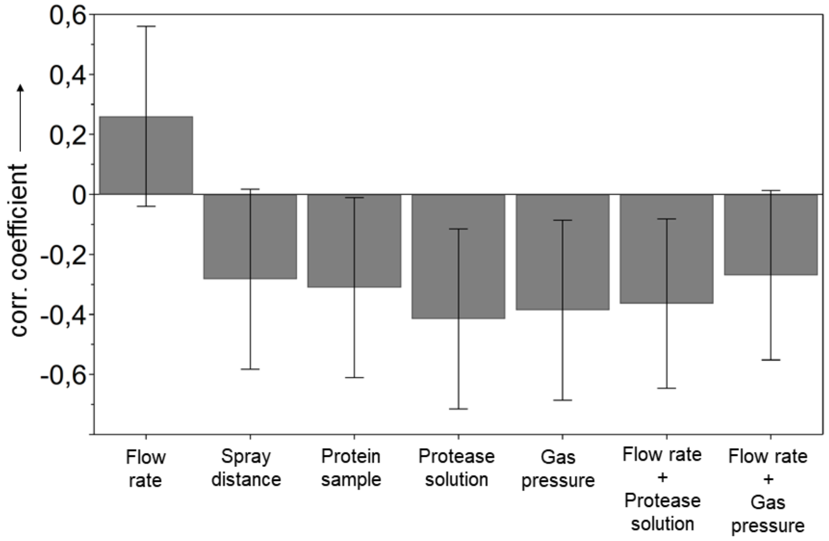

Figure S5. Result of DoE screening. Normalized coefficients plot for the output variable average peptide length obtained by an analysis of 19 randomized runs (R2 = 0.80, N = 19, DF = 11, confidence: 95%). For further details see the Experimental Section and Tables S3 and S5.

Table S4. DoE analysis wizard (MODDE ® Pro 12) information for the output variable sequence coverage.

Sequence coverage	Coeff. SC	Std. Err.	P	Conf. int(±)
Constant	46.6368	0.997194	4.67767e-12	2.25583
Flow rate	-0.954595	1.02452	0.375785	2.31764
Distance	5.66864	1.02452	0.000364334	2.31764
Sample solution	6.57609	1.02452	0.00012255	2.31764
Protease solution	8.03745	1.02452	2.58686e-05	2.31764
Gas pressure	-0.282843	1.02452	0.788726	2.31764
Flow rate*Gas pressure	6.72222	0.965926	6.61559e-05	2.1851
Distance*Sample solution	6.72222	0.965926	6.6156e-05	2.1851
Distance*Protease solution	5.34445	0.965926	0.000364334	2.1851
Sample*Protease solution	6.2	0.965926	0.000122551	2.1851
N = 19	Q2 =	0.691	Cond. no. =	1.061
DF = 9	R2 =	0.971	RSD =	4.347
Comp. = 1	R2 adj. =	0.942	Confidence =	0.95

Table S5. DoE analysis wizard (MODDE ® Pro 12) information for the output variable average peptide length.

Average peptide length	Coeff. SC	Std. Err.	P	Conf. int(±)
Constant	29.6037	0.342325	6.16342e-17	0.753442
Flow rate	0.671751	0.351705	0.0825427	0.774088
Distance	-0.729498	0.351705	0.062322	0.774088
Sample solution	-0.802567	0.351705	0.0433888	0.774088
Protease solution	-1.07245	0.351705	0.0110618	0.774088
Gas pressure	-0.997021	0.351705	0.0162316	0.774088
Flow rate*Protease solution	-0.94	0.331591	0.0162316	0.729817
Flow rate*Gas pressure	-0.695556	0.331591	0.0598451	0.729818
N = 19	Q2 =	0.547	Cond. no. =	1.061
DF = 11	R2 =	0.796	RSD =	1.492
Comp. = 1	R2 adj. =	0.666	Confidence =	0.95

Experimental Setup

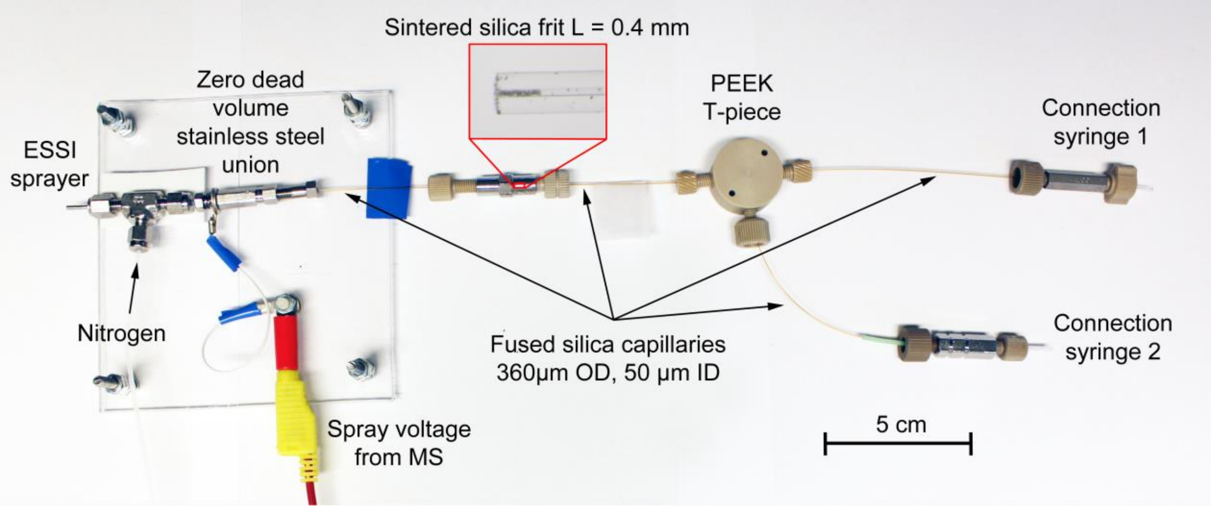

Figure S6. Experimental setup for accelerated pepsin digestion of proteins utilizing ESSI-MS. The ESSI spayer was orthogonally placed in front of the inlet of the mass spectrometer. The sintered frit was produced by tipping the tap of a fused silica capillary (360 µm OD, 50 µm ID) into Silica gel 60 for column chromatography (15 - 40 µm bead size; Merck, Germany). Sintering was performed by heating the packed section for approx. 10 seconds using a yellow glowing platinum wire. Excess silica beads were removed by flushing the capillary with deionized water.

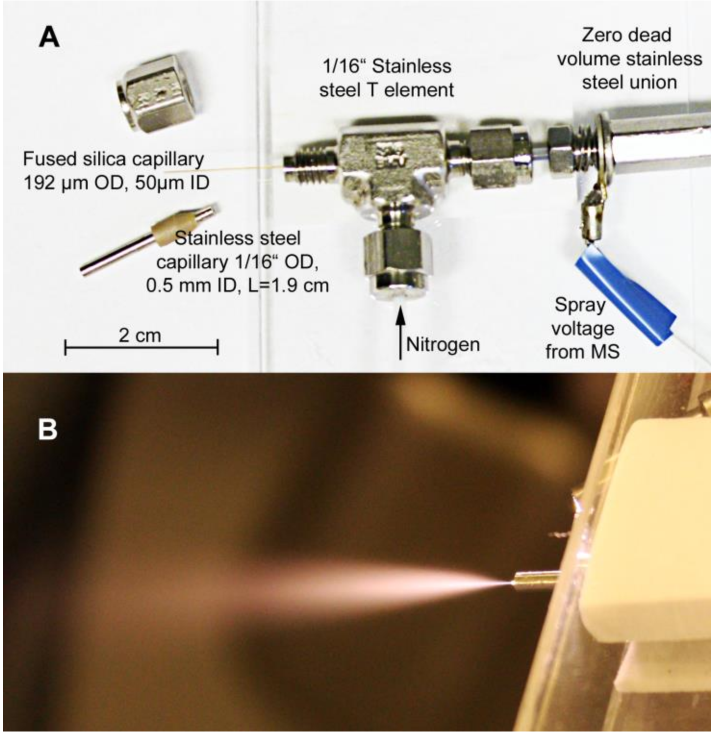

Figure S7. Top: Assembly of the modified ESSI sprayer according to Takáts et al.[3] Bottom: Photograph of the observed plume at the tip of the ESSI sprayer.

Control experiments

Bulk digestion of cytochrome c. A vial containing 50 µl of cytochrome c solution was heated to 95°C for 5 min for denaturation. After cooling to room temperature, 50 µl of the pepsin solution was added. The vial was mixed using a vortex mixer. After incubation for 3 hours at 37°C using a water bath, the sample was stored at -20°C until ESSI-MS analysis.

Quenching experiment. Experimental setup as depicted in Figure 1B. The two syringes were filled with protein solution *Cyt3* as well as protease solutions *Pep3*. 100 µl ammonia 0.5% quenching buffer (pH = 11.4 at 22°C) were placed in an Eppendorf vial on top of a vortex mixer. At a total flow rate of 2.5 µl·min^{-1} (1.25 µl·min^{-1} for each syringe) the stream of liquid was directed into the vial containing the quenching solution. Within 10 min 25µl of the eluting reaction mixture were collected in the quenching buffer, the final pH was checked with pH test strips and found to be approx. pH = 8.0. A 50 µl aliquot of the quenched solution was re-acidified with 10 µl of 8% formic acid (to final pH ~ 4.5) and submitted to ESSI-MS analysis. Considering the inner volume of a 16 cm, 50 µm i.d. capillary of approx. 0.3 µl, at a flowrate of 2.5 µl·min^{-1}, it takes the mixture around 7.5 seconds to pass the capillary from the mixing silica frit into the quenching solution, where the enzyme is irreversibly inactivated. Hence, we assume a reaction time for the digestion in solution of approx. 7.5 sec at a flow rate of 2.5 µL·min^{-1}.

References

(1) Chambers, M. C.; Maclean, B.; Burke, R.; Amodei, D.; Ruderman, D. L.; Neumann, S.;

Gatto, L.; Fischer, B.; Pratt, B.; Egertson, J.; Hoff, K.; Kessner, D.; Tasman, N.; Shulman, N.; Frewen, B.; Baker, T. A.; Brusniak, M.-Y.; Paulse, C.; Creasy, D.; Flashner, L.; Kani, K.; Moulding, C.; Seymour, S. L.; Nuwaysir, L. M.; Lefebvre, B.; Kuhlmann, F.; Roark, J.; Rainer, P.; Detlev, S.; Hemenway, T.; Huhmer, A.; Langridge, J.; Connolly, B.; Chadick, T.; Holly, K.; Eckels, J.; Deutsch, E. W.; Moritz, R. L.; Katz, J. E.; Agus, D. B.; MacCoss, M.; Tabb, D. L.; Mallick, P. A Cross-Platform Toolkit for Mass Spectrometry and Proteomics. *Nat. Biotechnol.* **2012**, *30* (10), 918–920. https://doi.org/10.1038/nbt.2377.

(2) Niedermeyer, T. H. J.; Strohalm, M. MMass as a Software Tool for the Annotation of Cyclic Peptide Tandem Mass Spectra. *PLoS One* **2012**, *7* (9), e44913. https://doi.org/10.1371/journal.pone.0044913.

(3) Takáts, Z.; Wiseman, J. M.; Gologan, B.; Cooks, R. G. Electrosonic Spray Ionization. A Gentle Technique for Generating Folded Proteins and Protein Complexes in the Gas Phase and for Studying Ion−Molecule Reactions at Atmospheric Pressure. *Anal. Chem.* **2004**, *76* (14), 4050–4058. https://doi.org/10.1021/ac049848m.
